# Trend in overweight and obesity among women of reproductive age in Uganda: 1995–2016

**DOI:** 10.1002/osp4.351

**Published:** 2019-07-11

**Authors:** S. Yaya, B. Ghose

**Affiliations:** ^1^ Faculté de Médecine Université de Parakou Parakou Benin; ^2^ Institute of Nutrition and Food Science University of Dhaka Dhaka Bangladesh

**Keywords:** Demographic and Health Survey, Overweight/obesity, Uganda, Women of reproductive age

## Abstract

Countries in Sub‐Saharan Africa (SSA) are experiencing rising burdens of overweight/obesity and associated non‐communicable diseases. As in other developing regions, this epidemiological transition in African countries is believed to be resulting from changes in dietary, sociodemographic structure and lifestyle factors. To date, not much is known about the prevalence and sociodemographic patterns of overweight/obesity in Uganda, especially among women of reproductive age. Therefore, this study aimed to address this research gap by using nationally representative data on women of this age group in Uganda.

**Methods:**

This study involved analysis of cross‐sectional data on 19,395 women aged between 15 and 49 years from Uganda Demographic and Health Survey for the years 1995–2016. Overweight/obesity was measured using body mass index as per World Health Organization guidelines, and logistic regression methods were used to identify the sociodemographic predictors.

**Results:**

There has a been significant rise in the prevalence of overweight (9.77% in 1995 vs. 16.21% in 2016) and obesity (1.99% in 1995 vs. 6.21% in 2016) since the first survey in 1995, with the most noticeable increase occurring in the central region that accounted for a combined prevalence of 17.22% in 1995 compared with 37. 21% in 2016. Multivariate analysis showed an increased likelihood of having overweight/obesity among women who live in the urban areas, have primary and above primary education, from non‐poor households and users of radio and TV.

**Conclusion:**

During the last two decades, there has been a slow but steady rise in the prevalence of overweight and obesity among women of reproductive age in Uganda. The present findings highlight the need for an enhanced attention on growing overweight/obesity within the broader goal improving maternal and child health in the country.

## Background

During the last two decades, there has been noticeable declines in maternal and child mortality across African region that is generally attributed to better access to maternal healthcare services and improved nutrition among women of reproductive age 1, 2. Between 1990 and 2013, children under 5 mortality declined at an average of 2.9% per year in Africa 3. The decline in historically high mortality rates and low life expectancy at birth, however, has been coincided with a slow and steady rise in overweight/obesity in the population, a phenomenon once believed to be characteristic of industrialized nations 4, 5, 6. A recent systematic review reported that between 1980 and 2014, the age‐standardized mean body mass index (BMI) increased from 21.0 to 23.0 kg m^−2^ in men and from 21.9 to 24.9 kg m^−2^ in women 5. The healthcare dilemma arising from the co‐existence of over‐nutrition and undernutrition, known as double burden of malnutrition, has been recognized as a significant public issue in several nations in Africa already 7, 8, 9, including some of the most impoverished ones such as Uganda 10.

The widening prevalence of excess body weight (overweight and obesity) particularly among women of reproductive age is a growing challenge for tackling maternal and child mortality owing to its association with increased risk of obstetric complications and infant mortality 11, 12, 13. Furthermore, maternal obesity is also a significant determinant of excessive weight gain among children 14. Given the already high rates of child mortality undernutrition in countries such as Uganda 15, 16, the rise in childhood obesity is particularly alarming because of its contribution to adult obesity and enhanced risks of developing non‐communicable diseases (NCDs) at later years. Making public health measures to tackle the spread of overweight and obesity and the associated risk factors is therefore of great public health importance.

The increasing rates of obesity, especially among the most impoverished communities, has been a subject of great research and policy concern. Previous studies have explained the aetiology of obesity from various genetic, behavioural, dietary, environmental, sociocultural and macroeconomic factors 17, 18, 19, 20, 21. Although genetic make‐up is considered a key aetiological factor, obesity is increasingly being understood as a complex interplay between a host of behavioural (dietary habit and physical activity), environmental (obesogenic environment, e.g. poor availability of fresh food and space for physical activity) and sociocultural factors (material well‐being, unequal distribution of privileges and political environment) 22, 23, 24. As the countries continue to urbanize with certain transformation in agriculture, dietary and sociocultural factors that are intimately linked with nutrition and body‐weight status, the situation of obesity is likely to worsen in years to come, unless appropriate measures are taken to control or reverse the trend.

Addressing overweight/obesity in a population that is characterized by widespread poverty, undernutrition and hunger is particularly challenging as the underlying pathways may not be the same among people at either ends of socioeconomic spectrum. Previous studies suggest that obesity is increasing in both the affluent and disadvantaged segments of the population and that the wealth–overweight/obesity relation is not a linear one 9, 19, 22. Individuals in impoverished families often tend to over‐eat to recover for prolonged episodes of food insecurity and usually rely on cheap sources of calories that are generally nutrient deficient and thus worsen nutritional imbalance. In contrast, overweight/obesity in the affluent families can be more common because of greater exposure to obesogenic factors such as habitual over‐eating, tobacco and alcohol use, greater consumption of meat, dairy and sugary products and decreased necessity for activities involving intense physical activity.

Overweight/obesity, similar to other chronic illnesses, is influenced by a range of demographic, environmental and socioeconomic factors 25, 26. Exploring the sociodemographic patterns is a key step towards developing a good understanding of the issue and employ population‐based prevention and intervention measures. To date, there is no nationally representative study assessing the situation of overweight/obesity in Uganda. To this regard, this study aimed to explore the trend and sociodemographic patterns of overweight and obesity among adolescent and adult women in Uganda. This study utilized open access data from Uganda Demographic and Health Survey (UDHS). Demographic and Health Survey (DHS) is a multicounty population health survey program that is operating in Uganda with the aim to provide quality data on key demographic and health indicators and thereby facilitating health monitoring and evidence‐based health policymaking in the population.

## Methods

### Setting

The Republic of Uganda is a landlocked nation East Africa sharing frontier with Kenya in the east, Tanzania in the south, Rwanda in the south‐west, the Democratic Republic of Congo in the west and Sudan in the north. Uganda became independent of British colonial rule in October 1962. The country has a population of 41.49 million (as of 2016) living in an area of 241,039 km^2^. Uganda is divided into 80 administrative districts, which are subdivided into counties, sub‐counties and parishes. The economy is based mainly on agricultural activities with coffee being the most important exporting product in terms of revenues. The country is generally food self‐sufficient and experienced a flourishing economy following independence. However, the country experiences long‐standing political violence and civil unrest with significant bearing on economic, social and healthcare infrastructure.

#### Data source

Data for this study were collected from DHS website that are available to registered users for research purposes. UDHS are conducted by Uganda Bureau of Statistics in collaboration with the Ministry of Health with technical and financial support provided by the Government of Uganda, the United States Agency for International Development, the United Nations Children's Fund and the United Nations Population Fund. The main purpose of these surveys is to provide countrywide data necessary for monitoring and evaluation of population, health and nutrition programmes and assist in evidence‐based health policymaking. The surveys are conducted by face‐to‐face interviews on eligible men (15–54 years) and women (15–49 years) using structured questionnaires containing several components: individual men, women, children (0–59 months), couples and households. Year of surveys and scope of sampling areas were listed in Table 1. Data for this study were based on women's questionnaire. More detailed version of the sampling techniques regarding the surveys were published in the final reports 27, 28, 29, 30.

**Table 1:** List of surveys used in the present study

Year	No. of clusters	Coverage	Field work	Response rate (%)
1995	303	All of the districts but Kitgum and Pader	March–August	95.8
2000–2001	298	All of the districts but Amuru, Bundibugyo, Gulu, Kasese, Kitgum and Pader	September 2000–March 2001	93.9
2006	368	All the districts	May–October	94.7
2011	404	All the districts	June–December	93.8
2016	697	All the districts	June–December	97

### Measures

#### Outcome variable

The outcome variable was overweight/obesity that was measured in terms of BMI by using the anthropometric measures (weight and height) provided on the UDHS data sets. DHS surveys collect anthropometric information among eligible and consenting women by using standard instruments.

#### Independent variables

Several sociodemographic predictors that are conceptually related with overweight/obesity were selected. The selection was facilitated by reviewing the past studies based on DHS data and was dependent on the availability of the variables on the data sets. Finally, the following were included in the analysis: age groups (15–19/20–24/25–29/30–34/35–39/40–44/45–49); residency (urban/rural); education (no education/primary/secondary and higher); religion (Christian, Islam/others); parity (0/1–2/>2); age at marriage (<18/18+); occupation (unemployed/technical/managerial/sales and others); wealth status (poor/non‐poor); radio use (no/yes); and TV use (no/yes). Operational definitions of these variables were provided in Table 2.

**Table 2:** Sociodemographic profile of the sample population

Variable	Definition	N = 19,395	%
Year	Year of conducting the survey		
1995		3,395	17.4
2001		5,793	29.7
2006		2,523	12.9
2011		2,341	12.0
2016		5,459	28.0
Age groups (27.88/9.13)	Age of respondents the time of survey		
15–19		4,173	21.4
20–24		4,029	20.6
25–29		3,498	17.9
30–34		2,736	14.0
35–39		2,225	11.4
40–44		1,664	8.5
45–49		1,187	6.1
Residency	Urbanicity of the residence		
Urban		3,691	18.9
Rural		15,820	81.1
Education	Experience of formal education		
No education		3,684	18.9
Primary		11,351	58.2
Secondary/higher		4,477	22.9
Religion	Religious affiliation of the respondent		
Christian		16,178	82.9
Islam/others		3,333	17.1
Parity			
Null		3,993	20.5
1–2		4,832	24.8
>2		10,686	54.8
Age at marriage	Age of first marriage of the respondent		
<18		8,658	56.4
18+		6,690	43.6
Occupation	Type of employment		
Unemployed		5,564	28.5
Technical/managerial		10,659	54.6
Sales/others		3,288	16.9
Wealth status	Based on household wealth quintile measured in terms of passion of durable goods^a^		
Poor		6,912	35.4
Non‐poor		12,600	64.6
Radio	Frequency of listening to radio		
No		4,581	23.5
Yes		14,931	76.5
Watches TV	Frequency of watching TV		
No		12,474	63.9
Yes		7,038	36.1

a DHS surveys measure wealth quintiles based on factor scores assigned for possession of durable goods (TV and refrigerator) that are used to categorize the households into quintiles such that lowest quintiles (poorest, poorer, middle, richer and richest) represent the poorest and the highest scores represent the richest households. For this study, poorer and poorest were categorized as *poor* and the rest of the quintiles as *non‐poor*.

### Data analysis

Data were analysed using STATA version 14 and SPSS version 24. Prior to analysis, the data sets were checked for missing values and outliers and then merged to perform pooled analysis. Collinearity tests were also run to diagnose the correlation between body weight and the covariates and to make sure none of the independent variables were strongly correlated. Following that, the data set was prepared for complex analysis by accounting for sampling weight, strata and primary sampling unit. Adjusting for sampling structure is recommended for DHS data because the surveys employ cluster sampling techniques. After preparing the plan file, chi‐square bivariate tests analyses were carried out to calculate the prevalence of overweight and obesity across the independent variables and to assess the significance of the associations. Trends in body‐weight status and regional prevalence were presented as bar and map charts. Finally, multivariate analysis was performed to examine the association between body‐weight status and the sociodemographic variables. This was done by using multinomial regression technique, and the outputs were presented as relative risk ratios (RRRs) and 95% confidence intervals (CIs). Three sets of regression models were run: univariate (unadjusted), full models (adjusting for all variables) and parsimonious models (adjusting for variables that were significant in the full model). Results of parsimonious models were interpreted as the main findings. Statistical significance was set at *p* < 0.05. Sensitivity analyses were performed by repeating the multivariate models for selected age groups at a time. Model fitness was assessed by Akaike information criterion and pseudo‐*R* ^2^ values.

#### Ethical clearance

All DHS surveys are approved by Inner City Fund (ICF) international and a review board in the implementing country. Institutional ethical approval was not necessary for this study as the data were secondary and were available in public domain in anonymized form.

## Results

### Sample characteristics

Sample characteristics are presented in Table 2. Mean age of the respondents was 27.88 years (SD = 9.13). Adolescent 15, 16, 17, 18, 19 and young 20, 21, 22, 23, 24 women represented more a two‐fifth of the total sample. Majority of the women were of rural residency (81.1%), had primary level education (58.2%), followers of Christianity (82.9%), had more than two children (54.8%), married before reaching 18 years (56.4%), employed in technical/managerial work (54.6%), from non‐poor households (64.6%), users of radio (76.5%) and user of TV (63.9%).

#### Prevalence of overweight/obesity

As shown in Table 3, the prevalence of overweight and obesity was 12.4% (95% CI = 11.7–13.1%) and 3.9% (3.6–4.4), respectively. The prevalence of overweight was higher among women aged below 30 years, whereas that of obesity was higher among those aged between 25 and 24 years. The prevalence of both declined gradually for age groups above 29 years and lowest among women in the age groups of 45–49. The prevalence was also noticeably higher among women of rural residency, had primary level education, followers of Christianity, employed in technical/managerial type of work and from non‐poor households. Regarding media use status, women who reported listening to radio and TV had significantly higher percentage of having obesity (*p* < 0.05) 31.

**Table 3:** Prevalence of overweight and obesity among non‐pregnant women in Uganda (n = 19,395)

	Normal weight 83.7% [82.8, 84.5]	Overweight 12.4% [11.7, 13.1]	Obese 3.9% [3.6, 4.4]	p‐values
Age groups				
15–19	21.7 [20.8, 22.5]	19.0 [17.2, 20.9]	14.0 [11.3, 17.3]	<0.001
20–24	21.8 [21.0, 22.6]	19.0 [17.2, 21.0]	13.1 [10.5, 16.1]	
25–29	17.9 [17.2, 18.6]	18.2 [16.2, 20.5]	18.4 [15.2, 22.0]	
30–34	14.0 [13.3, 14.6]	15.2 [13.6, 16.9]	16.4 [13.6, 19.6]	
35–39	10.9 [10.4, 11.5]	12.9 [11.1, 14.8]	15.2 [12.1, 18.9]	
40–44	8.3 [7.7, 8.8]	8.9 [7.7, 10.3]	12.8 [10.3, 15.8]	
45–49	5.6 [5.1, 6.0]	6.8 [5.8, 8.1]	10.2 [7.8, 13.3]	
Residency				
Urban	16.0 [14.5, 17.7]	31.1 [27.8, 34.6]	44.2 [38.9, 49.7]	<0.001
Rural	84.0 [82.3, 85.5]	68.9 [65.4, 72.2]	55.8 [50.3, 61.1]	
Education	21.2 [19.9, 22.5]	12.0 [10.4, 13.8]	11.7 [9.1, 14.8]	
No education	58.7 [57.4, 60.0]	56.1 [53.6, 58.6]	47.6 [43.0, 52.2]	<0.001
Primary	20.1 [18.9, 21.4]	31.9 [29.4, 34.5]	40.7 [35.8, 45.9]	
Secondary/higher	21.2 [19.9, 22.5]	12.0 [10.4, 13.8]	12.0 [9.1, 14.8]	
Religion				
Christian	83.0 [81.4, 84.5]	82.0 [79.8, 83.9]	80.1 [75.9, 83.8]	<0.001
Islam/others	17.0 [15.5, 18.6]	18.0 [16.1, 20.2]	19.9 [16.2, 24.1]	
Parity				
Null	19.4 [18.4, 20.4]	21.6 [19.7, 23.7]	18.2 [15.1, 21.7]	0.055
1–2	25.9 [25.0, 26.9]	24.1 [22.1, 26.3]	23.6 [19.7, 28.1]	
>2	54.7 [53.7, 55.7]	54.2 [51.8, 56.6]	58.2 [53.7, 62.5]	
Age at marriage				
<18	57.7 [56.5, 58.8]	50.5 [47.6, 53.4]	49.2 [44.3, 54.1]	0.041
18+	42.3 [41.2, 43.5]	49.5 [46.6, 52.4]	50.8 [45.9, 55.7]	
Occupation				
Unemployed	27.6 [26.1, 29.2]	29.9 [27.6, 32.3]	27.7 [24.0, 31.8]	<0.001
Technical/managerial	57.2 [55.3, 59.1]	48.3 [45.4, 51.3]	40.9 [36.4, 45.5]	
Sales/others	15.1 [14.2, 16.2]	21.7 [19.7, 24.0]	31.4 [27.4, 35.6]	
Wealth status				
Poor	36.5 [34.2, 38.9]	30.7 [27.7, 34.0]	24.3 [20.3, 28.7]	<0.001
Non‐poor	63.5 [61.1, 65.8]	69.3 [66.0, 72.3]	75.7 [71.3, 79.7]	
Radio				
No	26.2 [24.5, 28.1]	17.3 [15.4, 19.3]	11.2 [8.4, 14.9]	<0.001
Yes	73.8 [71.9, 75.5]	82.7 [80.7, 84.6]	88.8 [85.1, 91.6]	
Watches TV				
No	65.5 [62.8, 68.1]	60.0 [57.0, 63.1]	42.3 [37.3, 47.5]	<0.001
Yes	34.5 [31.9, 37.2]	40.0 [36.9, 43.0]	57.7 [52.5, 62.7]	

**Figure 1:**
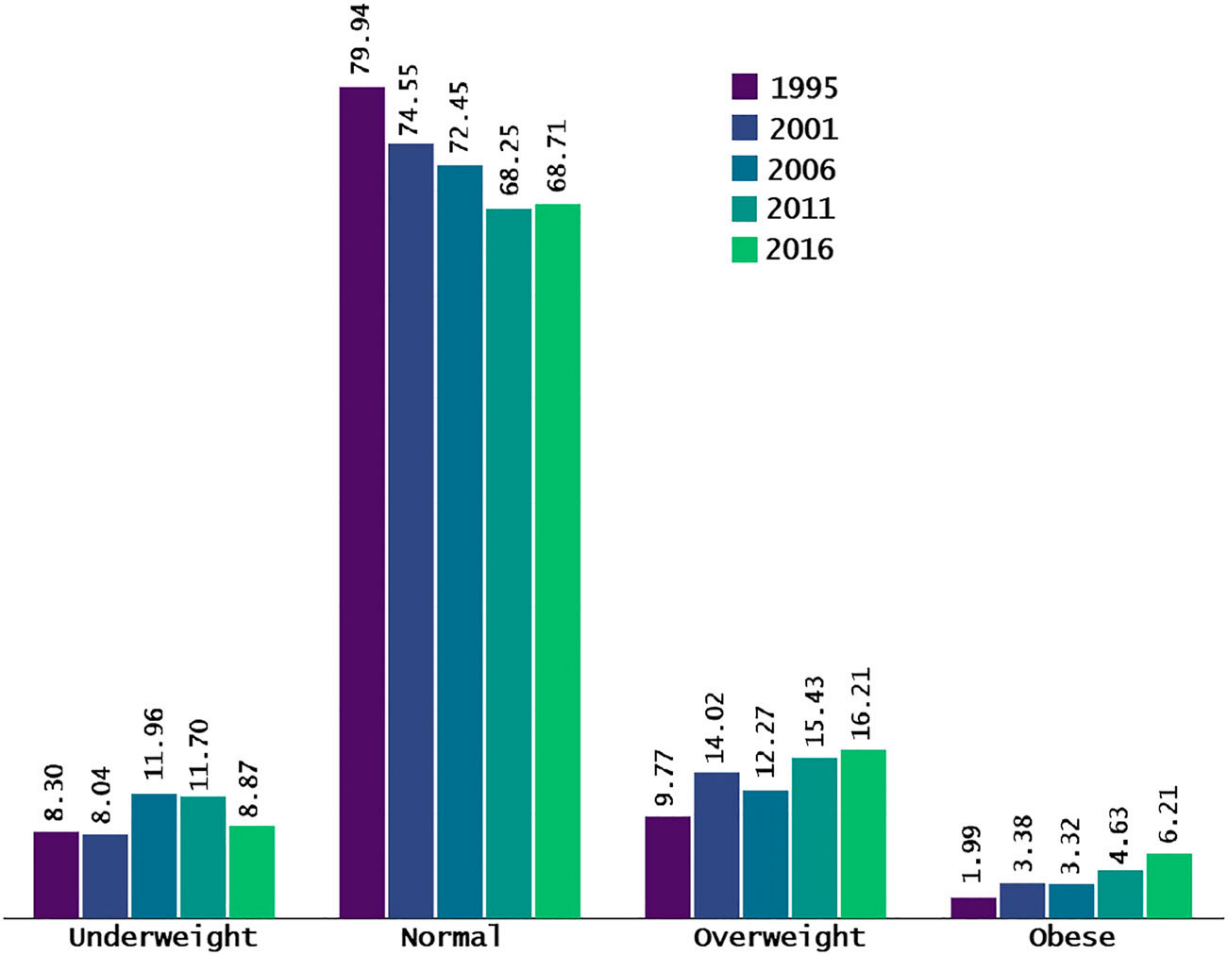
Trend in body mass index among women of reproductive age in Uganda. 1995–2016.

**Figure 2:**
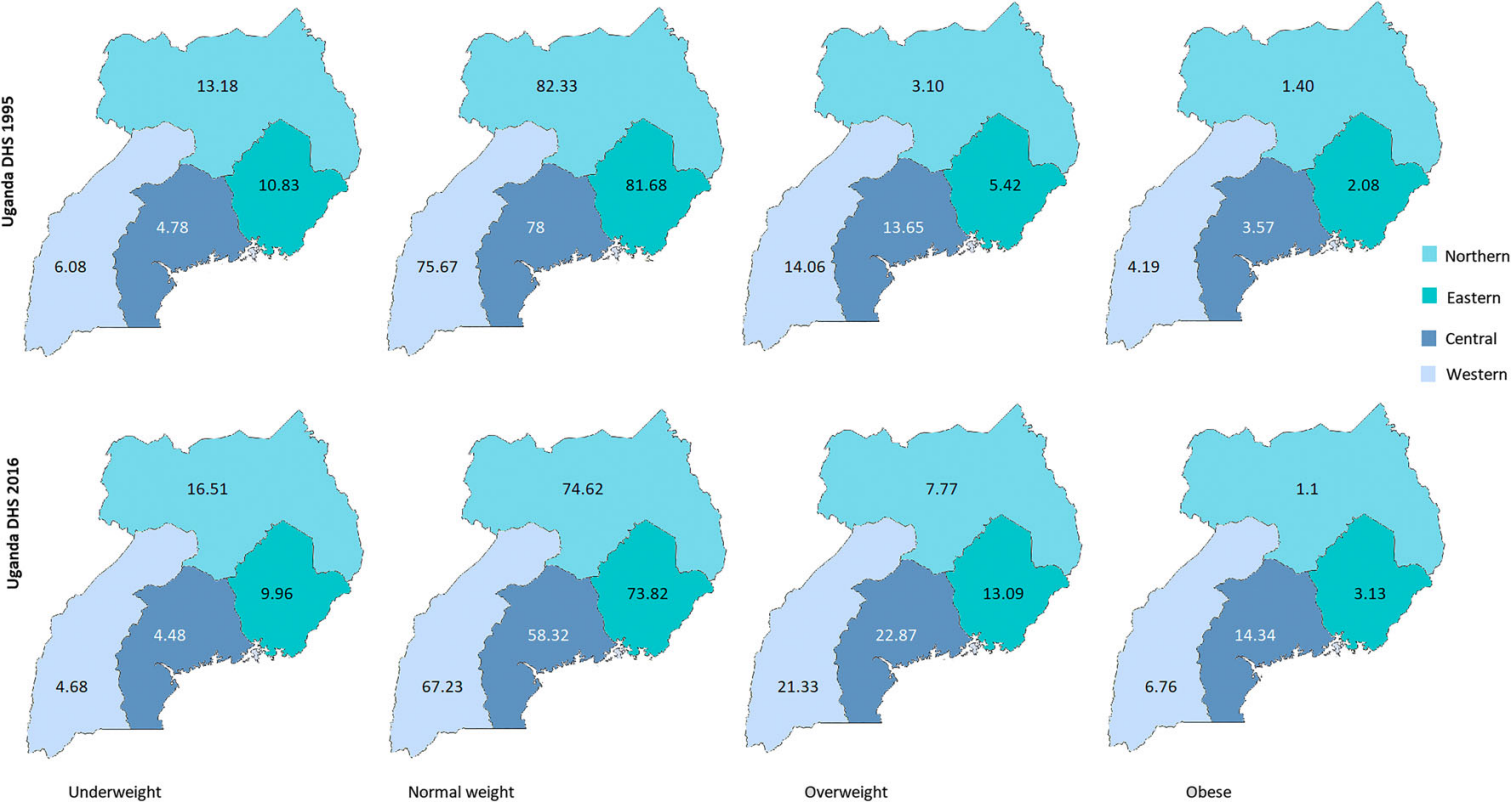
Regional disparities in body weight between 1995 and 2016.

Figure 1 and 2 respectively illustrates the trend in BMI status across the survey years and their regional distribution. From Figure 1, the prevalence of underweight women has remained virtually unchanged over the last two decades; however, those with overweight and obesity has increased by nearly twofold and threefold.

Figure 2 shows that between 1995 and 2016, the prevalence of underweight has slightly decreased in all the regions except the Northern where overweight has more than doubled during the same time. The figure further illustrates that in 2016, both overweight and obesity have become more prevalent in all four regions, except for obesity in Northern region. The change in body‐weight status across the surveys years was statistically significant (*p* < 0.05).

### Predictors of overweight/obesity among women in Uganda

Results of unadjusted and adjusted regression analyses were summarized in Table 4. In the full model, all the predictors variables showed significant association (*p* < 0.05) except for religion, parity and age at marriage.

**Table 4:** Factors associated with overweight and obesity among women of reproductive age in Uganda

	Univariate		Full model		Parsimonious	
	Overweight	Obesity	Overweight	Obesity	Overweight	Obesity
Year (1996)						
2001	1.616	1.838	2.803***	5.506***	2.654***	4.533***
	[1.401, 1.864]	[1.390, 2.431]	[2.329, 3.374]	[3.998, 7.582]	[2.245, 3.138]	[3.341, 6.151]
2006	1.351	1.828	2.553***	5.228***	2.237***	4.426***
	[1.137, 1.605]	[1.327, 2.519]	[2.059, 3.166]	[3.625, 7.540]	[1.846, 2.713]	[3.141, 6.238]
2011	1.964	2.437	2.495***	3.778***	2.664***	4.157***
	[1.667, 2.315]	[1.786, 3.325]	[2.044, 3.046]	[2.657, 5.373]	[2.229, 3.183]	[3.003, 5.754]
2016	2.112	3.520	3.156***	6.351***	3.160***	7.115***
	[1.835, 2.432]	[2.698, 4.592]	[2.646, 3.763]	[4.695, 8.592]	[2.692, 3.709]	[5.350, 9.463]
Age (15–19)						
20–24	1.100	1.043	1.176	1.364	1.194*	1.075
	[0.964, 1.254]	[0.801, 1.358]	[0.928, 1.492]	[0.815, 2.283]	[1.042, 1.368]	[0.818, 1.413]
25–29	1.246***	1.618***	1.473**	2.093**	1.407***	1.705***
	[1.090, 1.425]	[1.261, 2.076]	[1.146, 1.893]	[1.246, 3.517]	[1.222, 1.620]	[1.310, 2.218]
30–34	1.300***	2.064***	1.650***	3.172***	1.587***	2.508***
	[1.128, 1.498]	[1.607, 2.653]	[1.269, 2.147]	[1.872, 5.373]	[1.366, 1.844]	[1.920, 3.276]
35–39	1.352***	2.393***	1.823***	3.884***	1.716***	3.053***
	[1.163, 1.573]	[1.852, 3.093]	[1.394, 2.396]	[2.271, 6.644]	[1.463, 2.014]	[2.317, 4.023]
40–44	1.311***	2.363***	1.737***	3.703***	1.638***	2.873***
	[1.108, 1.551]	[1.789, 3.121]	[1.309, 2.305]	[2.133, 6.429]	[1.371, 1.957]	[2.129, 3.876]
45–49	1.318***	2.440***	1.824***	4.007***	1.730***	3.329***
	[1.090, 1.593]	[1.800, 3.310]	[1.354, 2.457]	[2.267, 7.086]	[1.415, 2.114]	[2.396, 4.624]
Residence (urban)						
Rural	0.477***	0.288***	0.562***	0.414***	0.637***	0.456***
	[0.437, 0.520]	[0.250, 0.332]	[0.498, 0.633]	[0.340, 0.504]	[0.574, 0.706]	[0.383, 0.542]
Education (none)						
Primary	1.691***	1.655***	1.566***	1.451**	1.564***	1.465**
	[1.482, 1.930]	[1.301, 2.107]	[1.356, 1.807]	[1.117, 1.885]	[1.363, 1.794]	[1.138, 1.886]
Secondary/higher	2.760***	3.751***	1.890***	2.099***	1.840***	1.878***
	[2.399, 3.176]	[2.934, 4.797]	[1.586, 2.252]	[1.557, 2.829]	[1.572, 2.153]	[1.424, 2.477]
Religion (Christian)						
Other	1.097	1.215***	1.020	0.992	NA	NA
	[0.985, 1.223]	[1.017, 1.452]	[0.898, 1.158]	[0.803, 1.227]		
Parity (null)						
1–2	0.932	1.028	0.841	0.748	NA	NA
	[0.826, 1.051]	[0.826, 1.278]	[0.646, 1.096]	[0.476, 1.176]		
>2	0.933	1.225***	0.829	0.760		
	[0.841, 1.035]	[1.017, 1.475]	[0.634, 1.084]	[0.486, 1.188]		
Age at marriage (<18)						
18+	1.260***	1.344***	0.968	0.853	NA	NA
	[1.147, 1.385]	[1.148, 1.574]	[0.872, 1.075]	[0.714, 1.018]		
Occupation (unemployed)						
Technical/managerial	0.732***	1.234***	0.919	1.037	0.887*	0.961
	[0.665, 0.806]	[1.103, 1.381]	[0.808, 1.045]	[0.825, 1.304]	[0.796, 0.989]	[0.788, 1.173]
Sales/others	0.728***	1.996***	1.074	1.414**	1.028	1.416***
	[0.611, 0.867]	[1.667, 2.390]	[0.935, 1.234]	[1.127, 1.774]	[0.912, 1.158]	[1.165, 1.721]
Wealth status (poor)						
Non‐poor	1.365***	1.880***	1.150**	1.303**	1.159**	1.374***
	[1.249, 1.490]	[1.600, 2.209]	[1.036, 1.277]	[1.079, 1.572]	[1.056, 1.271]	[1.156, 1.632]
Radio (No)						
Yes	1.797***	2.860***	1.558***	2.222***	1.600***	2.032***
	[1.606, 2.011]	[2.273, 3.600]	[1.359, 1.786]	[1.679, 2.940]	[1.420, 1.804]	[1.594, 2.590]
TV (No)						
Yes	1.435***	2.732***	1.726***	3.408***	1.612***	3.080***
	[1.320, 1.561]	[2.368, 3.152]	[1.525, 1.955]	[2.817, 4.122]	[1.453, 1.789]	[2.609, 3.636]
AIC			1.059		1.051	
Nagalekerke *R* ^2^			0.231		0.231	

Figures represent relative risk ratios. Reference categories are shown in round brackets. 95% confidence intervals in square brackets.

* *p* < 0.05.

** *p* < 0.01.

*** *p* < 0.001.

Women in the age group of 45–49 years had the highest likelihood of having overweight and obesity compared with those in the lowest age group. In the parsimonious model, however, the effects slightly attenuated but remained statistically significant, indicating that the relationship between age and body weight is influenced by other sociodemographic factors in the model. Urban women were found to have significantly high likelihood of being overweight [RRR = 0.637; 95% CI = 0.574, 0.706] and obese [RRR = 0.456; 95% CI = 0.383, 0.542] compared with their non‐urban counterparts. Religious affiliation, parity and age at marriage did not show any significant association with body weight. Participants employed in technical/managerial jobs had lower likelihood [RRR = 0.887*; 95% CI = 0.796, 0.989] of being overweight and those in the sales/other jobs had higher likelihood [RRR = 1.416; 95% CI = 1.165, 1.721] of having obesity. There was a significant dose–response relationship between educational attainment and body‐weight status, with the RRR of overweight being 1.564 [95% CI = 1.363, 1.794] and 1.840 [95% CI = 1.572, 2.153] times among with primary and secondary education compared with women having no formal education. Compared with women in the poor households, those in the non‐poor households were more likely to be overweight [RRR = 1.159; 95% CI = 1.056, 1.271] and obese [RRR = 1.374; 95% CI = 1.156, 1.632]. Listening to radio and TV watching were also associated with higher likelihood of having overweight and obesity. Women who reported listening to radio and watching TV had 2.032 [1.594, 2.590] and 3.080 times [2.609, 3.636] higher likelihood of having obesity.

The parsimonious models were repeated by stratifying by age groups to check whether it altered the associations between BMI and the sociodemographic variables. The stratified results showed no noticeable difference from the original models. The predictive capacity of the models was assessed by Nagalekerke *R* ^2^ values that indicated poor–moderate model fit (0.231). As both full and parsimonious model were used, Akaike information criterion was used to assess the quality of models and found that the parsimonious model showed a slightly better fit for the data.

## Discussion

The present study analysed nationally representative data from UDHS during the period between 1996 and 2016. Given the long‐standing burden of malnutrition and maternal and child mortality in the country, studies on body‐weight composition as a measure of nutritional status have significant policy implications. In short, the estimates indicate a significant change in body‐weight status among women of reproductive age. Since 1995, the prevalence of underweight has remained almost static, whereas that of overweight and obesity has increased by nearly twofold and threefold, respectively, indicating a higher relative rise in the obesity than overweight. The magnitude of the increase varied across the four regions. The most noticeable increase in overweight and obesity was observed in the central region that accounted for a combined prevalence of 17.22% in 1995 compared with 37. 21% in 2016.

Data on overweight/obesity among women of reproductive age in Africa are rare; however, this study found that the prevalence of obesity among of similar age group is comparatively lower in Uganda than other countries in East Africa including Kenya (20.3%) 32. The lower burden of overweight/obesity should be interpreted with caution as it can confound the magnitude of nutritional problems of the population. The results identify little less than a tenth of the women as undernourished, with no sign of progress during the last two decades. Persisting undernutrition among women of childbearing age is a critical public health concern 33 and therefore needs to be treated with urgency. Thus, based on the findings, it is recommendable that public nutrition programmes in Uganda focus not only on tacking the rising prevalence of overweight/obesity but also on the persisting prevalence of undernutrition among women of reproductive age.

Significant sociodemographic patterns were observed in the distribution of overweight and obesity. In general, women with higher age groups, urban residency, higher education, better household wealth status, higher parity, and access to electronic media had higher odds of being overweight and obese. Although weight gain tends to increase with age and the population in Uganda (as seen across the continent) is predominantly young, the protective effect of age structure can be offset by lower mean age at marriage and higher parity. This is particularly the case for women in Africa where physical activity is less common 34, 35, and the prevailing cultural norm embodies corpulence as a sign of wealth/fertility and a measure of beauty that enhance the prospect of marriage 36, 37, 38. Controlling overweight/obesity should focus on age‐specific interventions especially among women with higher parity.

The findings also indicated a positive effect of urbanicity and socioeconomic status on body weight. The mechanism through which urbanicity influences overweight is believed to be linked with dietary and lifestyle factors such as sedentary lifestyle, lower need for strenuous activity, high fat and sugar diet and more take away meals. Urban population also tend to have better access to educational and employment opportunities 39 that are also strongly correlated with economic well‐being and adoption of obesogenic lifestyle habits 40. As supported by the current findings, women with better education and wealth status are at greater likelihood of being oversized. The positive correlation between socioeconomic status and overweight/obesity poses a critical challenge as socioeconomic empowerment is regarded as a cornerstone of promoting women's health. Strategic health policymaking tailored to the local sociocultural circumstance is necessary to ensure a better coherence between health and socioeconomic well‐being among women.

Apart from the sociodemographic factors, media use status also appeared to be a significant predictor of overweight and obesity in this Ugandan women population. The media use–obesity relationship represents another paradoxical situation given the fact that media is regarded as a key instrument for public health communication and behavioural modification strategy 41, 42. In traditional Ugandan societies where women tend to have lower socioeconomic capacity and access to outdoor entertainment resources, electronic media such as TV and radio serve as a common form of pastime which in turn encourages sedentary behaviour 6, 40. Healthcare systems and policymakers need to consider these environmental conditions and create opportunities for more active lifestyle especially women, young women.

The present study has important policy implications. As a demography characterized by high rates of maternal and child malnutrition and mortality, rising maternal obesity can exacerbate the situation and compromise existing intervention programmes. Despite the growing recognition of the adverse impacts of maternal obesity on childbirth and infant survival outcomes, there has been no research so far on overweight/obesity among women of reproductive age. Introduction and implementation of policies to promote nutritional status among women require availability of reliable and up‐to‐date estimates of body weight over time. To this regard, the present study aimed to provide a detailed scenario of overweight/obesity prevalence across demographic and geographic parameters to inform policy approaches. As the study was based on existing data, the present analysis was unable to assess the contribution of several key determinants of excess body weight such as dietary and health behaviour, the sociocultural paradigms that encourage obesogenic habits and familial predisposition to obesity. Future studies should aim to addressing these limitations and focus on a broader range of sociocultural factors unique to Ugandan society. Sociocultural factors that trigger obesogenic behaviour (e.g. binge eating), stigma associated with seeking health care for obesity as well as gestational weight gain should be given special attention for future research on obesity and in making obesity‐prevention policies 43, 44, 45, 46. It is equally necessary to establish a national database for monitoring trends and progress towards nutrition‐related goals in the population.

This is the first study to report the temporal trend in body‐weight status among women of reproductive age in Uganda. Sample population were considerably large and nationally representative. Overweight and obesity were defined and categorized as per standard guidelines by World Health Organization. Data were analysed using rigorous methods to ensure robustness of the findings and reported in a manner to facilitate comparison with existing studies.

Besides the strengths, there are several important limitations to note. Firstly, DHS surveys do not collect information on dietary and lifestyle behaviour that are considered as the most proximal determinants of overweight/obesity. As such, it was not possible to adjust the analysis for behavioural factors that might have affected the strength or direction of the associations. Individuals from household in the higher wealth quintiles are supposed to have better access to healthy and nutritious food compared with those in the lower wealth quintiles and are more capable of controlling their caloric consumption and body‐weight status. Tobacco and alcohol consumption and physical activity patterns have also been shown to interact with dietary behaviour in determining weight gain among adult populations. Therefore, it would be interesting to explore the effects of sociodemographic factors on body‐weight measures segregated by dietary and lifestyle behaviour. It was also no possible to distinguish whether the overweight/obesity was pre‐gestational or because of weight gain from recent pregnancy. Women with higher parities usually experience greater weight retention and find it difficult to restore pre‐pregnancy body composition. However, this study attempted to minimize the effects of these limitations by adjusting the analysis for parity as well as by excluding the participants who were pregnant at the time of the survey. Apart from the selection of variables, there are limitations regarding the validity of the measures as most of the variables were in fact self‐reported (e.g. education, parity and age at marriage) and hence are subject to reporting/recall bias. Last but not least, the surveys were cross sectional and prevents making any causal inference.

## Conclusion

In this large‐scale cross‐sectional study based on nationally representative data from UDHS, this study found that the prevalence of overweight and obesity among women of reproductive age has been rising steadily during the last two decades. There is a strong demographic and socioeconomic pattern in the distribution of this growing burden that should be taken into consideration in designing effective intervention strategies. For countries like Uganda, addressing overweight/obesity in a population traditionally characterized by chronic hunger and undernutrition is a complex exercise. Targeted policies by identifying the culture‐specific risk factors as well as classifying the at‐risk populations can help controlling the dual burden of over‐nutrition and undernutrition in a context‐appropriate manner.

## Funding

The authors have not received any grant for this research.

## Conflict of Interest Statement

None declared.

## Ethics Approval

All DHS surveys are approved by ICF international and a review board in the implementing country.
